# Supplementary material for: Prognosis Communication in Pediatric Oncology: A Systematic Review
Source: Children (Basel). 2023 May 30;10(6):972. doi: 10.3390/children10060972 (PMC10297328; doi:10.3390/children10060972)
Supplement: Supplementary file 1 [file children-10-00972-s001.zip › children-2341449-supplementary.pdf]

Table S1. This table contains the most important findings from each of the included studies.

Nr.	Focus on	Most important findings
1 [7]	Parents and clinicians	-white parents were older and had higher education<br>-hispanics extremely or very upset in regards to child's likelihood of cure<br>-87% of parents wanted as much details as possible with no significant differences between race/ethnicity<br>-92% black parents wanted more details but physicians believed that only 25% wanted this level of detail<br>-parental race/ethnicity was associated with parent-reported peace of mind, with 50% of black parents and 47% of Hispanic parents reporting peace of mind relative to 26% of white parents
2 [8]	Parents	-185 discussions (92.5%) were recorded, totaling >3300 minutes of recorded medical dialogue, resulting in high acceptability rates for survey and interview<br>-longitudinal data were captured for 31 of 34 patient/parent dyads (91.2%)<br>-only 1 parent reported participation to be a "very" distressing experience<br>-the majority of parents described study participation as "somewhat" or "very" useful to them
3 [9]	Parents and children	-child age associated with presence: ages 3–6 and 7–12 less likely to be present than infants or adolescents<br>-children presence was more likely in solid tumors cases than children with hematologic malignancies<br>-children were less likely to be present if they had a physician-rated likelihood of cure of less than 90%
4 [10]	Parents	-most parents considered explicit sources (conversations with medical staff) "very" or "extremely" informative (73%-85%)<br>-implicit sources (parents' subjective opinions) were similarly informative (84%-87%)<br>-27% of parents reported prognostic estimates matching physicians' estimates<br>-parents who valued implicit information had lower prognostic accuracy (OR= 0.50; 95%CI: 0.29-0.88)<br>-parents were more likely to use implicit sources if they reported receiving high-quality prognostic information (OR= 3.02; 95%CI: 1.41-6.43), trusted the physician (OR= 2.01; 95%CI: 1.01-3.98) and reported high-quality physician communication (OR= 1.81; 95%CI: 1.00-3.27)
5 [11]	Parents and children	-nearly half of participants (patients = 48.9%, parents = 50.5%) displayed prognostic optimism compared with the determined objective estimate of curative potential<br>-the majority of patients (78%) and parents (85%) reported belief in a very high chance of cure, although fewer reported that their physician communicated a very high chance for cure (patients = 57%, parents = 70%)<br>-only 43% were determined to have a very high probability of cure<br>-significant differences were noted in prognostic optimism by cancer type ($P < .0001$); patients with solid tumor were more often optimistic, and patients with lymphoma were most often accurate
6 [12]	Parents	-among the parents of children with less favorable prognoses (<75% chance of cure), the receipt of high-quality information from the oncologist was associated with greater peace of mind (OR= 5.23; 95% CI: 1.81-15.16) and communication-

		related hope (OR= 2.54; 95%CI: 1.00-6.40)<br>-high-quality oncologist communication style was associated with greater trust in the physician (OR= 2.45; 95%CI: 1.09-5.48) and hope (OR= 3.01; 95%CI: 1.26-7.19)<br>-accurate prognostic understanding was less common among the parents of children with less than 50% chance of cure (OR= 0.39; 95%CI: 0.17-0.88).
7<br>[13]	Parents	-the majority of parents preferred to hear about prognosis in as much detail as possible (87%, 85%, and 84%, respectively, at the time of diagnosis, 4 months, and 12 months)<br>-the majority of parents reported having had new prognostic discussions with the physician at each time point (93%, 74%, and 81%, respectively, at the time of diagnosis, 4 months, and 12 months)<br>-85%, 87%, and 87%, respectively, at the time of diagnosis, 4 months, and 12 months of patients considered receiving a numeric estimate of prognosis to be extremely or very important<br>-irrespective of prognosis, parents were more likely to be satisfied with prognostic communication when physicians provided more extensive disclosure at the time of diagnosis (OR= 1.85 per element of disclosure; 95%CI: 1.25-2.74) and when physicians discussed prognosis again before 4 months (OR= 8.71; 95%CI: 2.64-28.72)
8<br>[14]	Parents	-clinicians presented prognostic information in all cases<br>-parents revealed what further information they desired and clinicians explained that large grade of variability in cure for high-risk brain tumors<br>-explicit communication about prognosis did not satisfy parents' desire for information about their own child; parents tried to personalize prognostic information
9<br>[15]	Children	-overall, participants wanted medical information to be provided to them by their healthcare providers and wanted to be direct participants in medical conversations<br>-many participants displayed some ambivalence or conveyed conflicting wishes for prognostic information, but most preferred the information first hand<br>-main sources of information were primarily clinicians, then parents and other sources<br>-almost every participant discussed social concerns as a key concern for their present and future life<br>-many wished for return to normalcy, had feelings of missing out<br>-some focused on getting better and trying not to consider other possible outcomes, while others were anxious about recurrence or death, and a few were in denial
10<br>[16]	Parents	-approximatively 46% of parents found information regarding future limitations to be extremely or very upsetting<br>-parents were more likely to consider information regarding future limitations distressing if they also found prognostic information upsetting (OR= 5.36; 95%CI: 3.34- 8.6), struggled to accept their child's illness (OR= 2.57; 95%CI: 1.53- 4.31), or had depression (OR= 1.79; 95%CI: 1.18- 2.72)<br>-parents who believed they played a passive role in<br>decision making were less likely to be upset by information regarding future limitations (OR= 0.52; 95%CI: 0.3-0.94)

		-approximately 92% of parents considered information regarding potential future limitations to be extremely/very important<br>-those who found information regarding future limitations distressing were significantly more likely to consider it important (96% vs 89%) and to desire a precise understanding of their child's risks (92% vs 80%)
11 [17]	Clinicians and general population	-92.7% of physicians said that children should be informed of their incurable illness, only 50.7% of the general population agreed<br>-physicians were also more likely to think that younger children should know about their poor prognosis compared with the general population<br>-physicians who opposed incurable illness disclosure suggested that children might not understand the situation, whereas the general public was primarily concerned that disclosure would exacerbate the disease<br>-physicians who were women or religious were more likely to want to inform children of their poor prognosis<br>in the general population, gender, education, comorbidity, and caregiver experience were related to attitude toward poor prognosis disclosure to children
12 [18]	Parents	-26 descriptors were given, resulting in 3 main themes: 'becoming aware', 'the changes' and 'being in the situation'<br>-the descriptors "dread", "dizzy", "sick", and "shocked" were linked to becoming aware of the disease<br>-the descriptors "heavy" and "numb" were linked to a deeper awareness and fuller understanding, during the later stages of the consultation<br>-3 descriptors were given as originating from the clinicians and their delivery of the news: "supported", "included", "trusting", with the feeling of "trust" involving the clinician's openness and honesty<br>-parents expressed feelings of "relief" and "gladness" when told of the terminal prognosis and the end of treatments with distressing side effects<br>-parents expressed fear for the children's pain and anxiety<br>-most parents felt "powerless" and "helpless"
13 [19]	Parents, children and clinicians	-40/265 recorded discussions took place at equivocal timepoints, comprising > 500 min of medical dialogue<br>-prognosis talk encompassed < 3% of dialogue and was absent in nearly half of equivocal discussions (17/40, 42.5%)<br>-curability statements were identified in only two conversations<br>-inductive content analysis of dialogue revealed four distinct patterns for communicating equivocal disease status: (1) up-front reassurance, (2) softening the message, (3) describing possible disease progression without interpretation, (4) expressing uncertainty without discussing the bigger picture
14 [20]	Parents and children	-a vast majority of parents wants information when their child's illness becomes<br>-fathers, reported less discussion time in this regard<br>-according to parents' reports, 87% of children received diagnostic information and only 44% (97/227) of the children received prognostic information
15 [21]	Parents	-most parents preferred shared decision making (64%), whereas 23% preferred parent-led decision making and only 13% preferred oncologist-led decision making<br>-parental decision-making preferences did not differ by race/ethnicity. However, the actual role parents played in decision making did, with 25% of white parents

		reporting parent-led decision making, versus 37% of black parents, 48% of Hispanic parents, and 56% of Asian/other parents<br>-oncologists accurately predicted parental preferences for decision making 49% of the time. Oncologists accurately predicted parental preferences for 53% of white parents, 23% of black parents, 37% of Hispanic parents, and 43% of Asian/other race parents
16 [22]	Parents	-8 distinct functions of communication in pediatric oncology were identified<br>-6 were similar to previous findings from adult oncology: (1) building relationships, (2) exchanging information, (3) enabling family self-management, (4) making decisions, (5) managing uncertainty, (6) responding to emotions<br>-2 functions not previously described in the adult literature were observed: (7) providing validation and (8) supporting hope. Supporting hope manifested as emphasizing the positives, avoiding false hopes, demonstrating the intent to cure, and redirecting toward hope beyond survival. Validation manifested as reinforcing "good parenting" beliefs, empowering parents as partners and advocates, and validating concerns<br>-although all functions seemed to interact, building relationships appeared to provide a relational context in which all other interpersonal communication occurred<br>-medical errors and inaccurate communication had a negative effect on this relationship, and parents expressed the importance of acknowledging mistakes
17 [23]	Parents	-71% of parents trusted the child's oncologist "completely" at baseline, as did 79% at 4 months and 77% at 12 months<br>-parents who completed all longitudinal assessments had higher educational attainment than those who participated only at baseline<br>-at baseline, high-quality physician communication (OR= 4.11; 95%CI: 1.78-9.51) and information (OR= 2.82; 95%CI: 1.29-6.16) were associated with trust, after adjustment for parent gender, race/ethnicity, and education<br>-parents were less likely to trust the physician completely at 12 months if the child had experienced cancer relapse or progression (OR= 0.28; 95%CI: 0.10-0.81)<br>-in a mixed linear model adjusted for parent gender, race/ethnicity, education, and clustering by physician, trust was associated with high-quality communication (OR= 3.40; 95%CI: 1.61-7.20) and receipt of high-quality information (OR= 2.48; 95%CI: 1.18-5.21), and inversely associated with relapse or progression (OR= 0.39; 95%CI: 0.17-0.92)<br>-trust increased over time
18 [24]	Parents	-only 26% of parents recognized that the chance of cure was <25%<br>-when asked to choose a single most important goal of care, approximately 72% chose cure, 10% chose longer life, and 18% chose quality of life; care goals were found to be associated with prognostic awareness, but not suffering from symptoms<br>-parents were more likely to prioritize quality of life when they recognized the child's poor prognosis<br>-approximately 41% of parents expressed regret about the most recent treatment decision; parents were more likely to experience regret if the child had received higher intensity medical care (OR= 3.14; 95%CI: 1.31–7.51), experienced suffering with limited benefit from the most recent treatment (OR= 4.78; 95%CI: 1.16–

		19.72), or experienced suffering from symptoms (OR= 2.91; 95%CI: 1.18–7.16)<br>-parents of children with poor-prognosis cancer frequently make decisions based on unrealistic expectations<br>-for a child who had no realistic chance of cure but was feeling well, approximately 48% of parents said a good parent should prioritize quality of life<br>-for a child who had no realistic chance of cure but was not feeling well, approximately 82% of parents said a good parent should prioritize quality of life<br>-the child's quality of life (PedsQL score >the median) was found to be inversely associated with parent reports of suffering with limited benefit from the last treatment, suffering from pain and suffering from any symptom
19 [25]	Clinicians	-6 levels of barriers to communication from the clinicians' perspectives were identified: individual, team, organizational, collaborating hospitals, community, and policy<br>-individual barriers subdivided into clinician characteristics, family characteristics, or characteristics of the clinician-family interaction; within each level and sub-level, we identified several manifestations of barriers.<br>-some barriers manifested similarly across professions and institutions (lack of comfort with difficult topics- individual, cultural differences- individual, lack of team shared mental model- team, or time pressure- organizational, while others manifested differently (need for boundaries- individual, intimidation or embarrassment of family- individual, unclear roles and authority- team, excessive logistical requirements- policy<br>-with the exception of "collaborating hospital," participants from all professions identified barriers from each level; physicians did not discuss collaborating hospital barriers
20 [26]	Parents, children and clinicians	-all oncologists thought that the announcement of the therapeutic futility places the parents in a psychological state of vulnerability that reduces their capacity of understanding and decision-making<br>-all parents spoke of having used the information provided by the oncologists in decision-making and of having accepted the recommendations of the oncologists without finding out thoroughly the risks and the benefits<br>-most parents deliberately decided not to inform their children of their terminal phase; some considered them to be too young or did not wish to cause their child additional pain<br>-all oncologists declared that palliative care should be proposed in case of lack of response to curative treatment; all oncologists declared that their role is one of "orienting" the choice of parents toward what they consider beneficial for the child<br>-most parents asked for professional interest, clear explanation of the situation, messages of hope<br>-all oncologists think that the adolescents should be made aware of their impending death<br>-many parents said that they preferred home as the place for end-of-life care, while some thought hospitalization was better or did not understand the terminal situation of the disease<br>-all the parents and adolescents had strong religious beliefs<br>-patients unaware of treatment futility prioritized cure and continuing to live and

		were encouraged so by their parents; aware patients wished for less suffering<br>-barriers: lack of prognosis understanding, emotional ties in parents, lack in psychology training for oncologists<br>-facilitators: disease progress and futility of treatment, parental firm decision, information access
21 [27]	Children	-5 super-ordinate themes were identified: 'initially I felt shocked and scared', 'chemo is an awful thing', 'please talk to me, the more I know the better I feel', 'I will accept treatment and quickly get used to it because I know<br>I will get better' and 'my family is vital'.

Table S2. Parents' demographic data.

Age		Sex		Education		Marital status		Race	
**Total**	**2705**	**Total**	**3120**	**Total**	**2825**	**Total**	**2926**	**Total**	**2878**
<30	283, 10.46%	Male	634, 20.32%	Some college or less	978, 34.62%	Married/ couple	2479, 84.72%	White	2250, 78.18%
30-39	1057, 39.08%	Female	2486, 79.68%	College/ professional school graduate	1847, 65.38%	Single/ divorced/ widowed/ other	447, 15.28%	African-American	218, 7.57%
40-49	1047, 38.71%							Hispanic	224, 7.78%
>50	318, 11.76%							Other	186, 6.46%
Missing *	8, 47.06%	Missing *	6, 35.29%	Missing *	8, 47.06%	Missing *	6, 35.29%	Missing *	4, 23.53%
NR *	5, 29.41%	NR *	2, 11.76%	NR *	5, 29.41%	NR *	4, 23.53%	NR *	5, 29.41%

*: % of 17 total studies with parental data; NR: not reported.

Table S3. Children demographics.

Age		Sex		Race		Presence at the initial discussion	
**Total**	**2628**	**Total**	**2714**	**Total**	**2469**	**Total**	**2329**
0-2	593, 22.56%	M	1378, 50.77%	White	1920, 77.76%	Yes	839, 36.02%
3-6	501, 19.06%	F	1336, 49.23%	African-American	187, 7.57%	No	1490, 63.98%
7-12	695, 26.45%			Hispanic	191, 7.74%		
13-18	839, 31.93%			Other	171, 6.93%		

Missing *	5, 29.41%	Missing *	6, 31.58%	Missing *	3, 15.79%	Missing *	0
NR *	0, 0.00%	NR *	2, 10.53%	NR *	8, 42.11%	NR *	9, 47.37%

*: % of 19 total studies with pediatric data; NR: not reported.

Table S4. Quality of life, number of recurrences/ relapses/refractory tumors and deceased patients

Quality of life		Recurrence/ relapse/ refractory tumor		Deceased	
**Total**	**439, 96.48%**	**250, 8.01%**		**232, 7.43%**	
Very good/ excellent/ low risk	211, 48.06%				
Good/ moderate risk	185, 42.14%				
Poor/ high risk	43, 9.79%				
NR *	16, 84.21%	14, 73.68%		11, 57.89%	
QD *	2, 10.53%				
NQD *	1, 5.26%				

*: % of 19 total studies with parental data; NR: not reported; QD: studies with quantifiable data; NQD: studies with unquantifiable data, studies that do not provide quantifiable data

Table S5. Physician demographic data.

Sex (n, %)		Experience/ role (n, %)	
Total	1372	Total	558
M	744, 54.23%	Primary/ attending physicians or >20 years experience	204, 36.56%
F	628, 45.77%	Specialist/ fellow physicians or <20 years experience	307, 55.02%
		Nurses/ nurse practitioners	28, 5.02%
		Other specializations	19, 3.41%
Missing *	1, 9.09%	Missing *	1, 9.09%
NR *	5, 45.45%	NR *	5, 45.45%

*: % of 11 total studies with physician data; NR: not reported.
